# Clinical Study of Oral Mucosal Lesions in the Elderly—Prevalence and Distribution

**DOI:** 10.3390/ijerph19052853

**Published:** 2022-03-01

**Authors:** Małgorzata Radwan-Oczko, Kinga Bandosz, Zofia Rojek, Joanna E. Owczarek-Drabińska

**Affiliations:** 1 Department of Oral Pathology, Wroclaw Medical University, 50-376 Wroclaw, Poland; malgorzata.radwan-oczko@umw.edu.pl; 2 Student Scientific Society of Oral Health, Department of Oral Pathology, Wroclaw Medical University, 50-376 Wroclaw, Poland; kinga.bandosz@student.umw.edu.pl (K.B.); zofia.rojek@student.umw.edu.pl (Z.R.)

**Keywords:** oral mucosa lesions, elderly, prevalence

## Abstract

Background: The aim of the study was to determine the prevalence, pattern, and distribution of oral mucosa lesions in elderly patients attending an outpatient oral pathology clinic. Methods: A retrospective analysis of 2747 patients’ medical files was performed. Of these, 1398 (50.6%) belonged to seniors. The mean age was 69.8 ± 7.1, and women were in the majority. Results: Among the entire group of patients, the three most common mucosal lesions were: oral lichenoid diseases (OLDs), lingual changes, and small nodules. In the female group, the most common diagnoses were: OLDs, lingual changes, and oral candidiasis; in the male population, the most common diagnoses were: lingual changes, small nodules, and OLDs. Those suffering from OLDs were the youngest patients, and those with focal oral mucosa erosions and ulcerations were the oldest patients. In the groups aged 60–70 and 71–80 years old, the most common diagnoses were OLDs, and in the oldest group (+80 years old), they were lingual lesions. Conclusions: Oral health has an obvious impact on the functional, psychological, and behavioral quality of life. There is a small number of publications concerning the prevalence and distribution of oral mucosa lesions in the senior population of European countries. Our study is filling in that void.

## 1. Introduction

Aging is a biological, irreversible process characterized by the loss of the physiological and functional integrity of organs and tissues. The general population is aging. People live longer, but they are subjected to long-lasting and persistent treatments of chronic systemic diseases. This trend is not just a current problem, but it will increase in the future along with the increase in the mean age of the population.

According to the World Health Organization (WHO), 60 years old is established as the turning point to “old age,” and demographic analyses show that the senior population will double by the year 2050.

Since the current social perspective of the beginning of “old age” is changing, Japanese geriatrics and gerontological societies have proposed new age categories for senescence. Thus, the years between 65 and 74 were labeled as “pre-old-age,” and years from 75 and above were described as “an old age” [1,2]. However, other countries and societies regard the threshold of old age as somewhere between the mid-40s to the 70s.

Therefore, the focus on the health and well-being of elderly people is growing, becoming a challenge for all clinicians in the near future.

The general health of the elderly is an important issue and it is inseparably related to oral health. The well-known statement that the oral cavity is “the mirror” of health makes sense. Some changes in oral mucosa can manifest different general pathological disorders such as: diabetes, skin diseases, immunological deficiencies and blood disorders, allergic and toxic reactions, gastric diseases, and vitamins and minerals deficiencies [3,4,5,6,7]. On the other hand, the association between systemic conditions (i.e., cardiovascular disease, diabetes, respiratory diseases, rheumatoid diseases, and periodontal disease) and oral health is well recognized and described [4,6,7].

Moreover, oral health has an obvious impact on the functional, psychological, and behavioral quality of life [8].

Current investigations suggest that in healthy subjects, morphological changes due to the aging process do not cause dramatic alterations in oral physiology, which generally stays intact in elderly people. From a clinical perspective, the oral mucosa of seniors may not have to differ from that of younger people. Nevertheless, the appearance of oral mucosa can change because of various long-standing factors in the oral cavity. Such factors include, among others, mechanical trauma and irritation, a lower level of saliva, mucosal-skin diseases, malnutrition, vitamin and microelements deficiencies, diabetes mellitus, hypertension, circulatory failure, cardiac diseases, gastrointestinal diseases, medications used to treat different diseases (both those with a general or local effect), the intake of tobacco and alcohol, as well as insufficient oral hygiene associated with different mental or physical limitations and the presence of specific dental disorders [9,10,11,12,13,14].

Furthermore, age-related changes manifesting in the oral cavity, such as: atrophy of oral mucosa epithelium, a lower level of mitotic activity and tissue regeneration, a reduction in cellular density, and the reduction in the amount of collagen and elastin in older patients should be taken into account [15]. These factors influence the environment of the oral cavity and facilitate irritation and damage to the oral mucosa epithelium. They can also change oral flora sensibility towards different viral, mycotic, or bacterial infections; hence, they are responsible for the presence of various lesions.

Thus, the oral cavity of the elderly exhibits a different environment when compared to that of younger subjects. Additionally, all mentioned contributing factors must be considered during the process of the diagnosis and treatment of oral mucosa lesions.

This study aimed to find the prevalence, pattern, and distribution of oral mucosa lesions in patients aged 60 years old and above who are attending an outpatient specialist oral pathology dental clinic.

## 2. Materials and Methods

The research was based on the retrospective analysis of the medical files of patients who attended an oral pathology outpatient clinic between the years of 2015–2019. The investigation of the medical files was performed by a dentist—a specialist in oral pathology. Trained and calibrated dentistry students, supervised by the same dentist who performed the review of the medical charts, took part in the analysis and data processing of the patients medical files. Diagnoses of the mucosal lesions, found in the medical charts, were made mainly on the basis of examination, observation, and clinical interview. When there was any doubt or necessity, additional diagnostic methods were used, including a smear with a microbiological examination, and a biopsy with full histopathology. Finally, a total of 2747 medical charts were screened. Patients below 60 years of age were excluded from the study; the use of fixed or removable dentures was not an exclusion criterion. Subjects aged 60 and older who consented to participate in the study were included in our research. Ultimately, 1398 medical records met the inclusion criteria. In the evaluation, in addition to the clinical diagnosis, gender and age were also taken into consideration. The informed consent was obtained from each patient or her/his legal guardians. No other personal data was collected or described. The approval of the local ethics committee was not required, since the data used was retrospective and there was no possibility of identifying an individual person from the data collected from the medical files.

### Statistical Methods

For each parameter, the mean (X), median (M), standard deviation (SD), range (min, max), and lower and upper quartile (25Q, 75Q) were calculated. The statistical significance between the means for different groups was calculated using one-way analysis of variance (ANOVA), alternatively using the non-parametrical Mann–Whitney U test (for two groups), or the Kruskal–Wallis test (for more than two groups), when the variances in groups were not homogeneous (the homogeneity of variance was determined using Levene’s test).

The statistical significance between the frequencies was calculated by the Chi-square test χ2df, with the corresponding degree of freedom df (df = (m − 1) × (n − 1), where m—number of rows, n—number of columns). The odds ratio (OR) and the 95% confidence interval for OR were calculated for 2 × 2 tables with statistical significance.

The relationship between the two parameters was assessed using correlation analysis and the Spearman correlation coefficients were calculated.

A *p*-value of less than 0.05 was required to reject the null hypothesis. Statistical analysis was performed using Epi Info Ver. 7.2.4.0 (Centers for Disease Control and Prevention (CDC) in Atlanta, GA, USA)and Statistica Ver. 13.3.(TIBCO Software, Palo Alto, CA, USA) software packages.

## 3. Results

The study investigated and only described the clinical diagnoses of oral mucosa lesions. Microscopic diagnoses of the lesions was always performed as a routine procedure when there were any doubts as to the correct initial diagnosis.

Among the total of 2747 medical files screened, 1398 (50.6%) belonged to senior patients (60 years or above).

The mean age was 69.8 ± 7.1, the median age was 68.0 (IQ range 64.0 ÷ 75.0), and the age range of the patients was between 60.0 and 93.0 years old. Women were in the majority, representing 73.7%, versus 26.3% represented by men (Table 1).

In the entire group of diagnosed patients, the most common mucosal lesions were: oral lichenoid diseases (OLD, according to the classification described by Aguirre-Urizar et al. [16,17]. OLDs included both oral lichen planus lesions (OLP) and oral lichenoid lesions (OLL), which may result from general medication intake. Both pathologies were diagnosed in white and white-red atrophic forms. OLD lesions were present in 18.3% of all of the patients and were statistically more frequent in women (21.0%) than in men (10.7%).

In 164 patients (11.8%), 108 women and 56 men, lingual changes (such as lingua geographica lingua villosa, lingua creanata, lingua fissurata, glossitis rhomboidea mediana, as well as pain at the tip of the tongue related to mechanical irritation and lingual varices) were diagnosed. Among these, the most frequently listed lingual lesion was geographic tongue (28.0%), followed by lingua villosa (21.3%). Lingual lesions were also statistically more frequently observed in women.

Different kinds of small nodules (fibromas, lipomas, epulis, and others) were the third most commonly diagnosed pathology (10.5% of the entire group). Contrary to OLDs, this pathology was statistically more common in men (12.8%) than in women (7.3%).

Candidosis, confirmed by laboratory tests, was diagnosed in 9.4%; it was also statistically more frequently observed in females (10.4%) than in males (6.6%) (Table 2).

The next most frequently observed lesions were burning mouth syndrome (BMS), observed in 7.9% of all patients, 8.3% and 6.8%, respectively, in women and men, and denture stomatitis, 6.8%, which was observed more often in men (8.2%) than in women (6.3%).

The following group of lesions, such as erosions and ulcerations of the oral mucosa, were present in 5.8% of the research group, 6.0% in females and 4.9% in male, respectively.

The least frequent pathology, present in at least 5% of our study group, was leukoplakia.

According to Shanbhah’s new proposed definition of leukoplakia [18], this lesion was defined when a predominantly white, irreversible, non-scrapable mucosal lesion could not be clinically or histopathologically characterized as any other lesion, was usually associated with the consumption of tobacco and/or alcohol, or was idiopathic. Both homogeneous and non-homogeneous lesions of leukoplakia were present in investigated patients.

This pathology was diagnosed in 5.5% of patients, with similar frequency in women (5.3%) and men (6.3%) (Table 2).

The youngest patients, with the median age of 66.0 years old, were found in the group diagnosed with oral lichenoid diseases (OLP and OLL).

**Figure 1:**
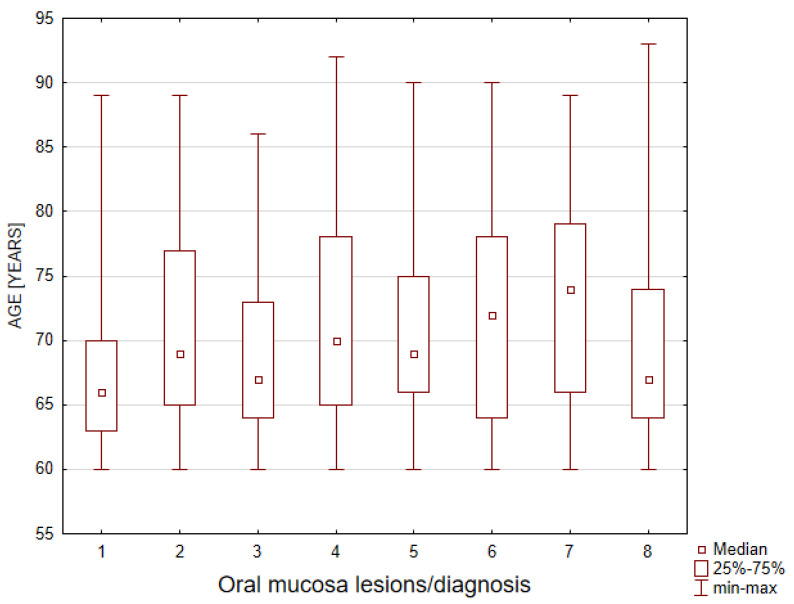
The age distribution of the patients in each oral mucosa diagnosis using the Kruskal- Wallis signed rank test, *p* = 0.000. The numbers found in the diagnosis axis refer to the diagnosis numbers from Table 3.

The oldest patients were subjects exhibiting various types of local erosions or ulcerations related to a local irritating factor (Table 3, Figure 1).

The eight most common diagnoses (featured in Table 3) comprised 1057 patients. In order to determine the frequency of the occurrence of mucosal lesions in relation to age, patients were categorized into three age groups: 60–70 years, 71–80 years, and 80+ years (Table 4). In the youngest and the most populated group (640 seniors), the most frequent were OLD lesions (30.0%), and the least frequent were local erosions and ulcerations (5.8%). In the middle group (317 patients aged 71–80 years), similarly, OLD lesions were dominant (18.0%), and the least frequent finding was leukoplakia, diagnosed in 5.0% of subjects. In the oldest and least populated group, the most common diagnosis was lingual lesions (18%). OLD lesions were the least frequent in this group, making up only 5.0% of the diagnoses.

In general, there were statistical differences in the frequency of oral mucosa lesions between these three age groups. However, oral leukoplakia was present in a very similar percentage between defined age groups: 8.0%, 5.0%, and 7.0%, respectively.

In 208 patients (15.0%), less-common diagnoses were observed. These oral mucosa lesions comprised from 1% up to 2.6% of all of the diagnoses. Among these were recurrent aphthae, xerostomia, morsicatio buccarum, angioma, angular cheilitis, mucocele, papilloma, hyperkeratosis, and granuloma fissuratum. Carcinoma in situ, confirmed by histopathological investigation, was diagnosed in five females and one male.

## 4. Discussion

So far, the prevalence of oral mucosa alterations and pathologies in the elderly has been discussed by only several studies from a few countries. In the available studies, assessed from using clinical and epidemiological methods, various influencing factors, such as: medications, metabolic changes, general health, malnutrition, geographical variations, and habits were taken into account [19,20,21]. Nevertheless, there is a small number of similar studies published for the senior population of some European countries.

The available, published results reveal a higher prevalence of oral mucosa lesions in the elderly compared to the middle-aged population. On the other hand, the presented results often conflicted with each other, due to differing variables and confusing factors that had not been taken into account in the study assessments. Additionally, it should be underlined that the senior population is not homogenous and that life expectancy, life attitude, life activity, daily behaviors and habits, mental states, or chronic physical disabilities vary from person to person [3,8,10].

This study presents the prevalence and distribution of oral mucosa lesions in patients aged 60 years and above. However, it should be noted that this evaluation was performed on the group of patients with symptomatic oral mucosa lesions, who came to the oral pathology clinic with an existing problem. Therefore, this is not a truly representative evaluation of the 60+ population, and our work focuses on the assessment of the type and age-related distribution of oral mucosa pathologies.

Females were the dominant group of our patients; therefore, the majority of the pathologies noted were present statistically more often in females than males. In the work of Rivera et al. [20], women were shown to be the most affected by OMLs, possibly because they are more concerned with their health and more focused on the prevention and quick diagnosis of any alteration, including those in oral cavity. Contrarily, Al-Maveri et al. found a statistically higher prevalence of OMLs in men [21].

In our study, a statistically higher prevalence was observed in men for diagnoses of: lingual changes, fibromas, epulis, and other small nodules. Denture stomatitis and oral leukoplakia were also more often found in men, but these were not statistically significant.

Oral lichenoid diseases (OLDs) were the most frequently observed lesion, including oral lichen planus (OLP) and oral lichenoid lesions (OLL). These two types of lesions were compiled together, due to the broad range of patient profiles. Some of the subjects suffered from hypertension, cardiac disease, and diabetes mellitus and may have been receiving specific treatments. Drugs used to treat the above-mentioned conditions are described in the literature as triggering factors of oral lichenoid lesions development. OLL can also mimic oral lichen planus. In two studies assessing the influence of cardiovascular drugs on the development of oral mucosa lesions, these lesions were found in 67.4% and 39.7% of patients, respectively [22,23]. Thus, in additional to idiopathic OLP, OLL lesions might also have been present in our research group.

Oral lichen planus it is a chronic inflammatory disease of unknown etiology, affecting mainly people aged 30–60 years, and women twice as often as men [24]. In turn, oral lichenoid lesions also occur as side effect of used medications. In our patients, the mean age in the group affected by OLDs was 67.2 years, and the mean duration of OLDs was 25 months. Thus, the OLDs could have started somewhere around the patients’ mid-forties, or later.

In our entire research group, OLD lesions were present in 18.3%, and presented more frequently in females. The group of patients diagnosed with OLDs was the youngest, and these pathologies were most often present in the youngest group (age range of 60–70), and the least often present in the oldest group (age range above 80).

In the group of patients aged 71–80 years, the most commonly found pathologies were focal mucosal erosions or ulcerations. The mean age of subjects suffering from this pathology was 72.4 years old. Ulcers developed due to local irritating factors (e.g., badly fitted dentures) and to the use of local and general medications are commonly observed in the oral cavities of seniors. The consumption of spicy foods, as well as certain types of vegetables and fruits, can result in the exacerbation of ulcer pain or soreness [22,25].

Various lingual lesions were diagnosed in 11.8% of the patients, lingua geographica and lingual villosa being the most common. The majority of these lesions did not require any treatment, but their etiology and management must be precisely explained to the patient to avoid false diagnosis and unnecessary or even improper treatment, which can change the oral cavity environment, possibly causing harm.

Other disorders, such as: recurrent aphthae, xerostomia, morsicatio buccarum, angioma, angular cheilitis, mucocele, papilloma, hyperkeratosis, and granuloma fissuratum were present at a very low prevalence of 2.6% to 1.0%. Among these lesions only angioma, mucocele, papilloma, hyperkeratosis, and granuloma fissuratum were more often diagnosed in men, but no statistical significance was observed.

Other diagnosed lesions in less than 1.0% of all of the investigated medical files included desquamative gingivitis, stomatitis herpetica, smoking-associated melanosis, cheilitis exfoliativa, leukoedema, Fordyce’s granules, contact allergy, and Heck’s disease.

In similar research, of 75 patients aged 55–90 years visiting a dental college in India, the authors found oral mucosa lesions associated with tobacco intake to be significantly more prevalent than lesions not related with nicotinism. The most common were: oral submucous fibrosis, smoker’s palate, and leukoplakia, as well as tobacco pouch keratosis [19].

A hospital-based, cross-sectional study of the Indian population revealed a high prevalence of oral mucosal lesions at a level of 59.6%. In this study, 750 geriatric participants, aged 60 years and above, were recruited. In the study group, men were twice as numerous as women. OMLs were significantly associated with the age group over 65, of male gender, with the presence of deleterious habits and denture use [25].

What must be emphasized is that the types of oral mucosa lesions, their prevalence, causes, prevention, and treatment vary according to country, society, and geographical region. Thus, it is important to be aware of these dissimilarities.

In the systemic review from thirteen countries, the most frequently observed OMLs in the elderly population were: denture-related stomatitis, present in 13.3%, irritation fibroma, observed in 8.7%, and fissured tongue, diagnosed in 6.3% of the studies. However, the more frequently described pathologies were: traumatic ulcerations, presented in 11 out of 15 articles, and oral lichen planus, discussed in 10 articles, followed by irritation fibromas, recurrent aphthous stomatitis, and melanotic pigmentation [21].

In a study of the Danish senior population, 75% of all of the 668 examined individuals displayed one or more oral mucosa lesions. Furthermore, such lesions were also present in 70% of seniors in the non-medicated group. The most prevalent were: lingual varicosities (seen mainly in patients with cardiovascular diseases), denture stomatitis, candidosis, (associated with an older age), fissured tongue, and frictional keratosis [13].

Furthermore, there is often a problem with the uncontrolled self-administration of the over-the-counter medications among seniors. Such long-term self-treatment can lead to the development of OMLs and should always be taken into consideration during the medical interview and diagnostic process for oral mucosal lesions.

Leukoplakia lesions, in many patients, were related to smoking. Because of a known close association between this pathology and smoking, these leukoplakias were treated in accordance to the existing protocols, with the elimination of the smoking habit, and the next step of management with a biopsy investigation and the determination of necessary further treatment.

In the case of idiopathic leukoplakia diagnosis, microscopic evaluation was consid- ered to assessed the possibility of the presence of dysplasia, thus allowing for the proper determination of further treatment.

Among the investigated patients, there were also salivary glands disorders. For the final suitable diagnosis and treatment, patients were referred to a maxillofacial surgical practice.

Currently, people are living longer, so the general medical examination of seniors should always include an oral cavity examination to prevent oral diseases by eliminating the factors that cause the pathologies of oral mucosa [26,27].

Meticulous oral hygiene, with proper brushing and the use of mouthwashes that do not contain irritating and drying agents (such as alcohol or specific herbs), appropriate prosthetic care, and treatment with artificial saliva substitutes are the first and basic factors in maintaining the health of the oral mucosa in the elderly.

It should also be emphasized that cooperation with general practitioners is crucial [4] and may bring measurable results in improving both oral and general health.

The elderly population is more likely to see general practitioners than dentists, in order to treat many general chronic diseases. Therefore, GPs should always remember to ask their patients about problems with their oral mucosa and, if possible, to try to examine the oral cavity.

## 5. Conclusions

The present study has provided observations about various aspects and types of oral mucosa lesion prevalence in older people—aged 60+. The most frequent pathologies were oral lichenoid diseases—OLDs. The majority of oral mucosa lesions were more often observed in women than in men. Potentially malignant lesions (oral leukoplakia) was significantly more often diagnosed in groups of younger seniors (aged 60–70 years).

Although this study provides helpful and important information on the incidence, gender, and age-related frequency of oral mucosa lesions in seniors, it has a few weaknesses. One is that the diagnoses were made in a group of patients who already had OML and presented with problems or concerns concerning their symptoms. Moreover, the presence of general diseases or the use of medications and supplements in the individual group members were not recorded. Furthermore, the number of oral cavity sites involved and the duration of the mucosal pathologies were not analyzed in this study. Despite these limitations, the results of the current research of the prevalence OMLs in a specific population group, namely the elderly, is useful in providing more information about its extension and characteristics. That knowledge is mandatory for the improvement of oral health promotion and prevention programs for specific age groups, as recommended by the World Health Organization, especially considering the aging of the global population [28].

## Figures and Tables

**Table 1:** Demographic characteristics of the group.

	Number of Patients	Median Age (Years)
Whole group	1398	68.0 (60.0–93.0)
Women	1025 (73.7%)	68.0 (60.0–93.0)
Men	366 (26.3%)	69.0 (60.0–93.0)

**Table 2:** The frequency of oral lesions in connection with gender.

Oral Mucosa Lesion/Diagnosis	Females		Males		χ^2^	*p*	OR	95% CI
	N	%	N	%				
OLDs n = 254	215	21.0	39	10.5	18.6	0.00002	2.26	1.55 ÷ 3.20
Lingual changes n = 164	108	10.5	56	15.3	5.44	0.0197	0.652	0.461 ÷ 0.922
Fibroma, lipoma, epulis, and other small nodules n = 146	93	9.1%	53	14.5%	7.83	0.00514	0.589	0.411 ÷ 0.845
Oral candidiasis n = 131	107	10.4	24	6.6%	4.32	0.0377	1.66	1.05 ÷ 2.63
BMS n = 110	85	8.3%	25	6.8%	0.604	0.437		
Denture stomatitis n = 95	65	6.3%	30	8.2%	1.18	0.277		
Focal erosions or ulcerations n = 80	62	6.0%	18	4.9%	0.445	0.505		
Leukoplakia n = 77	54	5.3%	23	6.3%	0.356	0.551		

**Table 3:** The median and mean ages of patients with oral mucosa lesions.

Oral Mucosa Lesion/Diagnosis	Mean Age (Years)	N	SD	MIN	MAX	25Q	M	75Q
1. OLD lesions	67.2	254	5.6	60.0	89.0	63.0	66.0	70.0
2. Lingual changes	70.6	164	7.2	60.0	89.0	65.0	69.0	77.0
3. Fibroma, lipoma, epulis, and other small nodules	68.7	122	5.9	60.0	84.0	64.0	67.0	73.0
4. Oral candidosis	71.4	131	7.4	60.0	92.0	65.0	70.0	78.0
5. BMS	70.9	110	7.0	60.0	90.0	66.0	69.0	75.0
6. Denture stomatitis	71.8	95	8.1	60.0	90.0	64.0	72.0	78.0
7. Focal oral mucosa erosions or ulcerations	72.4	80	7.5	60.0	89.0	66.0	74.0	79.0
8. Leukoplakia	69.2	77	7.6	60.0	93.0	64.0	67.0	74.0

Kruskal–Wallis test, *p* = 0.000.

**Table 4:** The frequency of oral mucosa lesions in the three age groups.

Oral Mucosa Lesion/Diagnosis	60–70 Years Women—491 Men—149		71–80 Years Women—227 Men—90		80+ Years Women—71 Men—29	
	N	%	N	%	N	%
1.OLDs n = 254	191	75.0%	58	22.5%	5	6.2%
Frequency	30%		18%		5%	
2. Lingual lesions n = 164	95	58.0%	51	31.0%	18	11.0%
Frequency	15%		16%		18%	
3. Fibroma, lipoma, epulis, and other small nodules n = 146	95	65.0%	43	29.0%	8	5.0%
Frequency	15%		14%		8%	
4. Oral candidiasis n = 131	66	50.0%	48	37.0%	17	13.0%
Frequency	10%		15%		17%	
5. BMS n = 110	66	60.0%	30	27.0%	14	13.0%
Frequency	10%		9%		14%	
6. Denture stomatitis n = 95	42	44.0%	37	39.0%	16	17.0%
Frequency	7%		12%		16%	
7. Focal oral mucosa erosions or ulcerations n = 80	31	39.0%	34	43.0%	15	19.0%
Frequency	5%		11%		15%	
8. Oral leukoplakia n = 77	54	70.0%	16	21.0%	7	9.0%
Frequency	8%		5%		7%	

χ^2^_14_ = 72.7, *p* = 0.00000. Patients 60–70 vs. 71–80 years, χ^2^_7_ = 36.5, *p* = 0.00001. Patients 60–70 vs. >80 years, χ^2^_7_ = 52.6, *p* = 0.00000. Patients 71–80 vs. >80 years, χ^2^_7_ = 15.5, *p* = 0.0305.

## Data Availability

The data that support the findings of this study are available from the corresponding author, J.E.O.-D., upon reasonable request.

## References

[1] Orimo H., Ito H., Suzuki T., Araki A., Hosoi T., Sawabe M. (2006). Reviewing the definition of ‘elderly’. Geriatr. Gerontol. Int..

[2] Ouchi Y., Rakugi H., Arai H., Akishita M., Ito H., Toba K., Kai I., on behalf of the Joint Committee of Japan Gerontological Society (JGLS) and Japan Geriatrics Society (JGS) on the definition and classification of the elderly (2017). Redefining the elderly as aged 75 years and older: Proposal from the Joint Committee of Japan Gerontological Society and the Japan Geriatrics Society. Geriatr. Gerontol. Int..

[3] Mehrotra V., Devi P., Bhovi T.V., Jyoti B., Pradesh U. (2010). Mouth as a mirror of systemic diseases. Gomal J. Med. Sci..

[4] Maeda K., Mori N. (2020). Poor oral health and mortality in geriatric patients admitted to an acute hospital: An observational study. BMC Geriatr..

[5] Cintra L.T.A., Samuel R.O., Prieto A.K.C., Sumida D.H., Dezan-Júnior E., Gomes-Filho J.E. (2017). Oral health, diabetes, and body weight. Arch. Oral Biol..

[6] Nazir M.A. (2017). Prevalence of periodontal disease, its association with systemic diseases and prevention. Int. J. Health Sci..

[7] Károlyházy K., Arányi Z., Hermann P., Vastagh I., Márton K. (2018). Oral Health Status of Stroke Patients Related to Residual Symptoms: A Case-Control Epidemiological Study in Hungary. Oral Health Prev. Dent..

[8] Gil-Montoya J., de Mello A.L.F., Barrios R., Gonzalez-Moles M.A., Bravo M. (2015). Oral health in the elderly patient and its impact on general well-being: A nonsystematic review. Clin. Interv. Aging.

[9] Daley T.D., Armstrong J.E. (2007). Oral manifestations of gastrointestinal diseases. Can. J. Gastroenterol..

[10] Yeh C., Katz M., Saunders M. (2008). Geriatric dentistry: Integral component to geriatric patient care. Taiwan Geriatr. Gerontol..

[11] Grimoud A., Lodter J., Marty N., Andrieu S., Bocquet H., Linas M.D., Rumeau M., Cazard J. (2005). Improved oral hygiene and Candida species colonization level in geriatric patients. Oral Dis..

[12] Yoshida M., Suzuki R., Kikutani T. (2014). Nutrition and oral status in elderly people. Jpn. Dent. Sci. Rev..

[13] Pedersen A.M.L., Nauntofte B., Smidt D., Torpet L.A. (2015). Oral mucosal lesions in older people: Relation to salivary secretion, systemic diseases and medications. Oral Dis..

[14] Capodiferro S., Limongelli L., Favia G. (2021). Oral and maxilla-facial manifestations of systemic diseases: An overview. Medicina.

[15] Rivera C., Arenas-Márquez M.J. (2017). Gerodontology: Effects of ageing on the oral mucosa. Rev. Clínica Periodoncia Implantol. Y Rehabil. Oral.

[16] Aguirre-Urizar J., Alberdi-Navarro J., de Mendoza I.L.I., Marichalar-Mendia X., Martínez-Revilla B., Parra-Pérez C., Juan-Galíndez A., Echebarria-Goicouria M. (2020). Clinicopathological and prognostic characterization of oral lichenoid disease and its main subtypes: A series of 384 cases. Med. Oral Patol. Oral Y Cir. Bucal.

[17] Urizar J.M.A. (2008). Letter to the editor: Oral lichenoid disease. A new classification proposal. Med. Oral Patol. Oral Y Cir. Bucal.

[18] Shanbhag V.K.L. (2017). New definition proposed for oral leukoplakia. Dent. Res. J..

[19] Patil S., Doni B., Maheshwari S. (2015). Prevalence and Distribution of Oral Mucosal Lesions in a Geriatric Indian Population. Can. Geriatr. J..

[20] Rivera C., Droguett D., Arenas-Márquez M.J. (2017). Oral mucosal lesions in a Chilean elderly population: A retrospective study with a systematic review from thirteen countries. J. Clin. Exp. Dent..

[21] Al-Maweri S.A., Al-Sufyani G.A., Tarakji B., Shugaa-Addin B., Al-Jamaei A.A. (2015). Oral mucosal lesions in elderly dental patients in Sana′a, Yemen. J. Int. Soc. Prev. Community Dent..

[22] Arunkumar S., Kalappanavar A., Annigeri R., Shakunthala G. (2013). Adverse oral manifestations of cardiovascular Drugs. J. Dent. Med. Sci..

[23] Mutafchieva M.Z., Draganova-Filipova M.N., Zagorchev P., Tomov G.T. (2018). Oral Lichen Planus—Known and Unknown: A Review. Folia Med..

[24] Jayakaran T.G. (2014). The effect of drugs in the oral cavity—A review. J. Pharm. Sci. Res..

[25] Cheruvathoor D.D., Thomas V., Kumar N.R., Jose M. (2020). High prevalence of oral mucosal lesions in elderly: Call for revolutionizing geriatric dental care strategies. J. Fam. Med. Prim. Care.

[26] Singh M.L., Papas A. (2014). Oral Implications of Polypharmacy in the Elderly. Dent. Clin. N. Am..

[27] Razak P.A., Richard K.M.J., Thankachan R.P., Hafiz K.A.A., Kumar K.N., Sameer K.M. (2015). Geriatric Oral Health: A Review Article. J. Int. Oral Health.

[28] Petersen P.E., Estupinan-Day S., Ndiaye C. (2005). WHO’s action for continuous improvement in oral health. Bull. World Health Organ..

